# Comparative Analysis of the Wood Metabolites of Three Poplar Clones Using UPLC-Triple-TOF-MS

**DOI:** 10.3390/molecules28207024

**Published:** 2023-10-11

**Authors:** Liping Li, Yun Liu, Xiaorui Yu, Xiaoqin Yang, Sida Xie, Guolei Zhu, Ping Zhao

**Affiliations:** 1 Key Laboratory of State Forestry and Grassland Administration on Highly Efficient Utilization of Forestry Biomass Resources in Southwest China, Southwest Forestry University, Kunming 650224, China; liuyun@swfu.edu.cn (Y.L.); yangxiaoqin@swfu.edu.cn (X.Y.); dream102035@163.com (S.X.); guoleizhu@163.com (G.Z.); 2 Yunnan Key Laboratory of Plateau Wetland Conservation, Restoration and Ecological Services, Southwest Forestry University, Kunming 650224, China; 3 Key Laboratory for Forest Resources Conservation and Utilization in the Southwest Mountains of China, Ministry of Education, Southwest Forestry University, Kunming 650224, China; tsxmr01@163.com

**Keywords:** wood, *Populus*, genetic background, comparative metabolomics

## Abstract

Poplar, a woody tree species, is widely used for industrial production and as a protective forest belt. Different clones of poplar exhibit clear variation in terms of morphological and physiological features, however, the impact of the genetic variation on the composition and abundance of wood metabolite have not been fully determined. In this study, ultra-high pressure liquid chromatography-triple time of flight-mass spectrometer (UPLC-Triple-TOF-MS) was used to explore the metabolite changes in poplar wood from three clones, including *Populus deltoides* CL. ‘55/65’, *P. deltoides* CL. ‘Danhong’, and *P. nigra* CL. ‘N179’. A total of 699 metabolites were identified. Clustering analysis and principal component analysis display that the metabolic differences of wood have allowed distinguishing different species of poplar. Meanwhile, eight significantly different metabolites were screened between *P. deltoides* and *P. nigra*, which may be considered as valuable markers for chemotaxonomy. In addition, the highly discriminant 352 metabolites were obtained among the three clones, and those may be closely related to the distinction in unique properties (e.g., growth, rigidity and tolerance) of the poplar wood cultivars. This study provides a foundation for further studies on wood metabolomics in poplar, and offers chemotaxonomic markers that will stimulate the early screening of potentially superior trees.

## 1. Introduction

Wood is usually termed the secondary xylem and synthesized in many plant species including short-lived annual species and long-lived perennials [1]. As the product of cambial cell differentiation [2], wood is rich in various metabolites, including those involved in primary metabolism (e.g., amino acids, lipids and carbohydrates) and secondary metabolism (e.g., alkaloids, terpenoids, polyphenols and tannins), which play a prominent part in plant growth, development and defence against abiotic and biotic stresses [3,4,5,6,7,8,9,10]. Due to the inherent physical properties and chemical component, wood is widely used for raw material of papermaking, building, chemical industry and even foods [11,12]. Furthermore, it is also a precious source of energy, as the renewable biofuels of solar-powered storage, and one of the primary carbon pools in terrestrial ecosystems, that has played an essential part in human life, industrial production and environmental fields [1,13,14,15,16].

Poplar (*Populus* spp., Salicaceae), one of the fast-growing pioneer trees with high biomass, contains more than 100 species, which are found widely distributed throughout in warm temperate and subtropical zones [17,18]. This plant has been long utilized in the commercial production and as a protective forest belt, making it useful in wood production and ecosystem maintenance [19]. However, numerous internal and external factors influence the yield and quality of poplar wood, including genetic origin, a variety of biotic (e.g., pathogen infection and insect attack) and abiotic (e.g., cold, heat, flooding, drought and salinity) stresses [5,9,20,21,22,23,24,25,26]. Therefore, based on the study of the molecular and biochemical mechanisms that control wood formation, various genetically modified poplar clones with the particular traits (e.g., adapted to specific environmental stresses, improved wood properties) are bred that will undoubtedly play a critical role in the economics and ecology in woody species.

Metabolomics, a powerful tool, can provide detailed explanation of the mechanisms behind various environmental (abiotic/biotic) and/or genetic stresses biological responses by high-throughput, non-biased and comprehensive profiling of the metabolite alterations in specific organisms or environmental conditions [27,28,29,30]. Currently, metabolomics strategies that combine different sample-processing technologies with various MS-based analytical platforms are used extensively for plant metabolomics analysis to achieve adequate coverage of the complex metabolite compositions from an organism [31] and provide highly efficient separation, identification and quantitative analysis of metabolites [7,28,32,33].

Currently, metabolomics studies on poplar clones are scarce and most of the analysis target is leaves of poplar in studies [24,34,35]. Herein, the metabolite compositions in woods across three different poplar clones were detected using ultra-high pressure liquid chromatography-triple time of flight-mass spectrometer (UPLC-Triple-TOF-MS) (Applied Biosystems, Waltham, MA, USA), and the distinctions of metabolite constitute were compared. The purpose was to understand the wood metabolites of the three poplar clones, explore the impact of the genetic variation on the composition of wood metabolite, and screen potentially useful chemotaxonomic markers that were identified to possibly be biomarkers for poplar genetic response, so as provide a scientific reference for future studies of genetic structure and early screening of potentially superior poplars.

## 2. Results

### 2.1. Metabolite Identification of Poplar Wood Based on UPLC-Triple-TOF-MS

To evaluate the metabolomic distinction of poplar wood from three clones, we conducted untargeted metabolic profiling to analyse the metabolome of wood from three poplar clones by using UPLC-Triple-TOF-MS.

**Figure 1:**
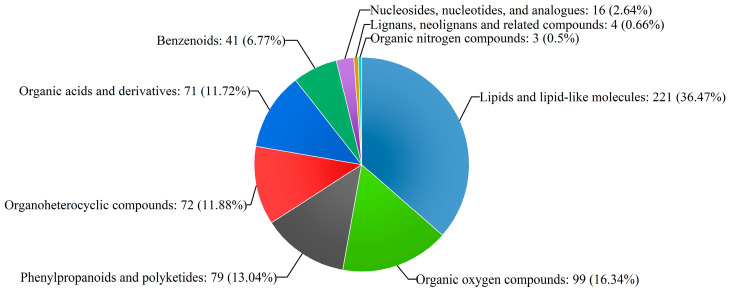
Classification of the identified metabolites.

After analysing the data matrix generated, 699 metabolites were predicted in poplar wood from three clones by comparing the accurate *m*/*z* values, associated adduct and MS/MS fragmentation data with those of the reference standards found in the databases (Supplementary Materials Table S1). According to annotations in human metabolome database (HMDB) (http://www.hmdb.ca/ (15 September 2021)), 606 metabolites were matched into 9 superclasses, including 221 lipids and lipid-like molecules, 99 organic oxygen compounds, 79 phenylpropanoids and polyketides, 72 organoheterocyclic compounds, 71 organic acids and derivatives, 41 benzenoids, 16 nucleotides and their derivatives, 4 lignans, neolignans and related compounds, and 3 organic nitrogen compounds (Figure 1). These metabolites were part of most of the primary and secondary metabolisms of poplar.

### 2.2. Principal Component Analysis (PCA) and Hierarchical Cluster Analysis (HCA) of Poplar Wood from Three Clones

**Figure 2:**
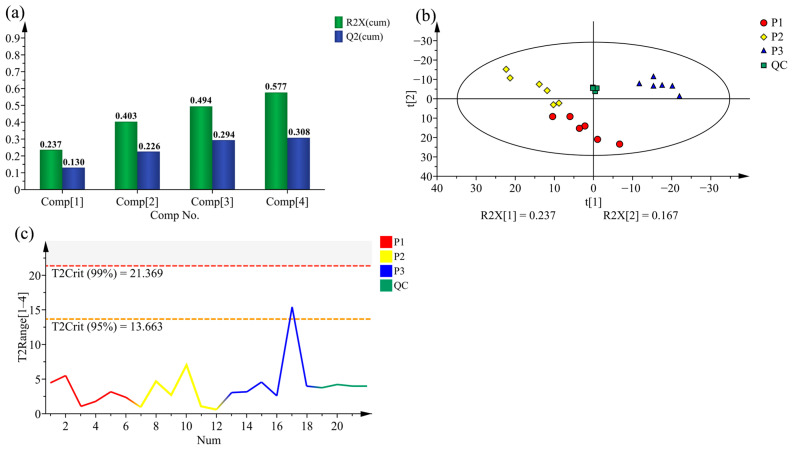
Principal component analysis (PCA). (**a**) Summary of fit. (**b**) PCA score plots of the metabolites in poplar wood from three clones, with quality control. (**c**) Hotelling’s T2 range line plot of all samples.

For the preliminary assessment about the differences between metabolic profiles of different poplar clones, PCA was conducted on the metabolic data for *Populus deltoides* CL. ‘55/65’ (P1), *P. deltoides* CL. ‘Danhong’ (P2) and *P. nigra* CL. ‘N179’ (P3). The analysis results demonstrated that the first four significant PCs can explain 57.7% of the overall variability, and PC1 and PC2 explain variation of 23.7% and 16.7%, respectively (Figure 2a). The first two PCs in the PCA plot (Figure 2b) display the three tested clones clearly clustered into two distinct chemotype-groups, indicating their corresponding classification of species. The clone P1 and P2 clustered together, resulting in rather weak explain variances, that was clearly separated from P3, indicating that the metabolites from P3 are remarkably different from both of those from P1 and P2. In addition, Hotelling’s T2 analysis for identified sample outliers display that the observations almost were distributed within the 95% confidence regions, reflecting the observations were available (Figure 2c) and the PCA model presented in this study was stable and reliable.

**Figure 3:**
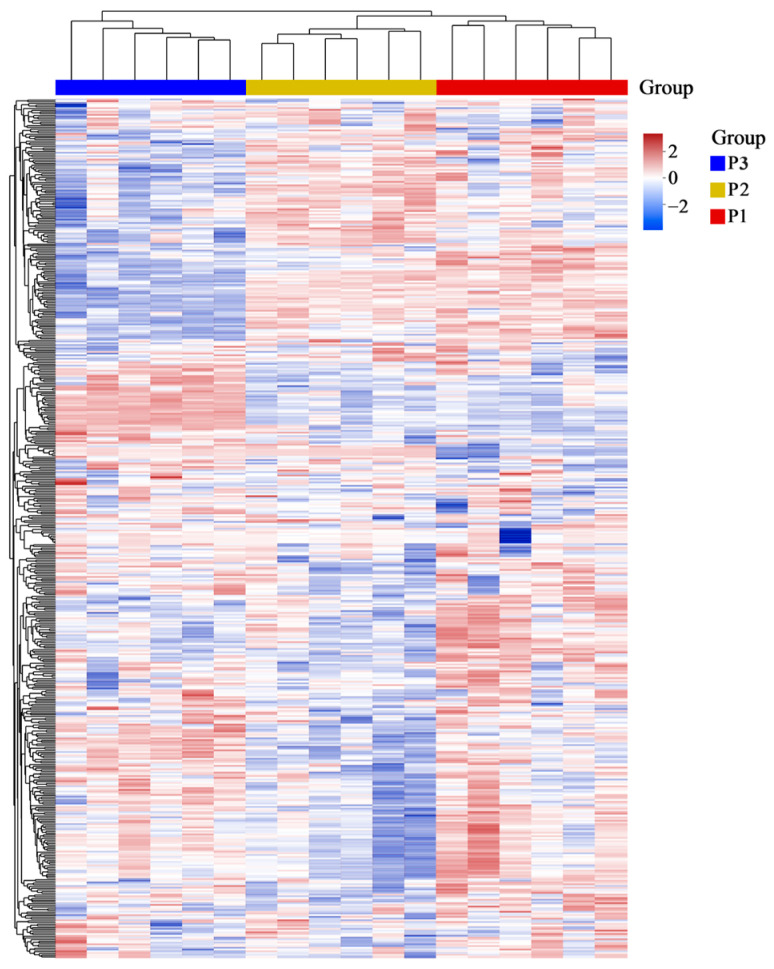
Cluster heatmap of the metabolites in poplar wood from three clones using HCA. Each column represents a sample, and each row represents a metabolite. Different colours represent the significant differences in the relative contents of metabolites between the different poplar clones, red bars indicate high contents, yet blue bars represent low relative contents.

In addition, HCA of the metabolites of the poplar wood from three clones was carried out. All identified metabolites are shown in Figure 3, substantial differences were observed in terms of the relative amounts of metabolites between the different poplar clones. Moreover, three clones were clearly separated into two main clusters by species, namely, *P. nigra* and *P. deltoides*. All samples that were initially characterized as P3 were clustered, while the other cluster was formed by two clones of P1 and P2. The clustering result indicated the expression profile of metabolites between two *P. deltoides* (P1 and P2) was similar, while showing relatively obvious difference with *P. nigra* (P3). The result is supported by PCA.

### 2.3. Supervised Orthogonal Partial Least Squares Discriminate Analysis (OPLS-DA) for All Samples

**Figure 4:**
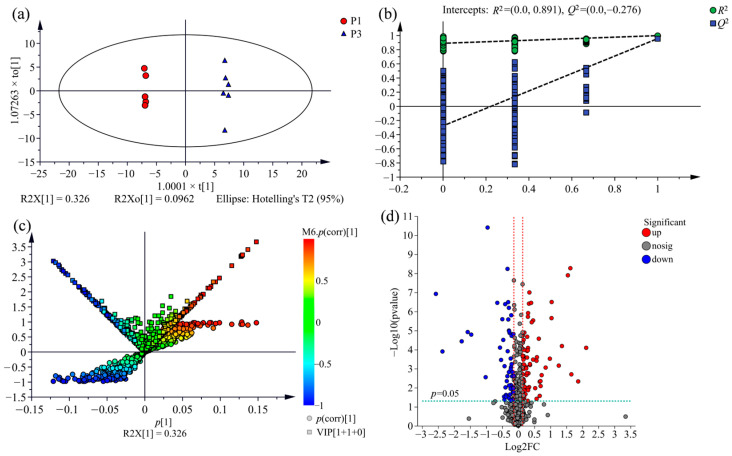
Differential metabolites analysis for the comparison group of P1 vs. P3 using OPLS-DA. (**a**) OPLS-DA score plot (R2X = 0.422, *R*^2^ = 0.999, *Q*^2^ = 0.957). (**b**) Permutation test plot with 200 iterations. (**c**) OPLS-DA (V + S)-plot, the potential differential metabolites situated on the lower left and upper right end of the “S”, the same are the upper of both side of the “V”, the deeper the colour of dots the more important metabolites. (**d**) Volcano plot. Each dot represents an identified metabolite; the blue dots on the left, the red dots on the right and the grey dots on the middle of diagram indicate down-regulated, up-regulated and insignificant differential metabolites, respectively.

**Figure 5:**
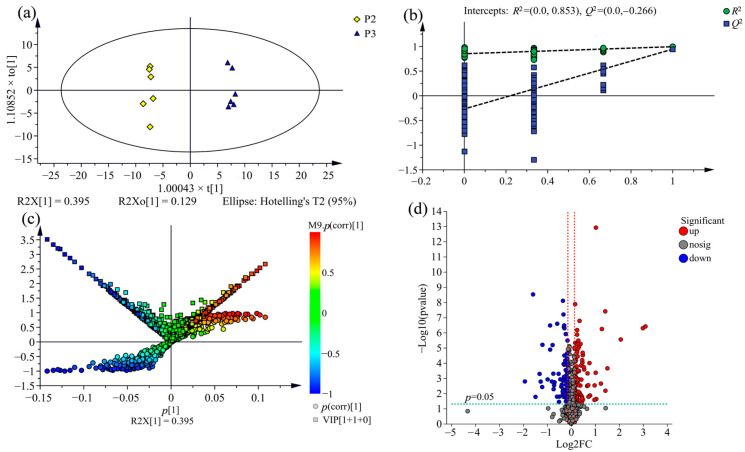
Differential metabolites analysis for the comparison group of P2 vs. P3 using OPLS-DA. (**a**) OPLS-DA score plot (R2X = 0.524, *R*^2^ = 0.995, *Q*^2^ = 0.945). (**b**) Permutation test plot with 200 iterations. (**c**) OPLS-DA (V + S)-plot, the potential differential metabolites situated on the lower left and upper right end of the “S”, the same are the upper of both side of the “V”, the deeper the colour of dots the more important metabolites. (**d**) Volcano plot. Each dot represents an identified metabolite; the blue dots on the left, the red dots on the right and the grey dots on the middle of diagram indicate down-regulated, up-regulated and insignificant differential metabolites, respectively.

**Figure 6:**
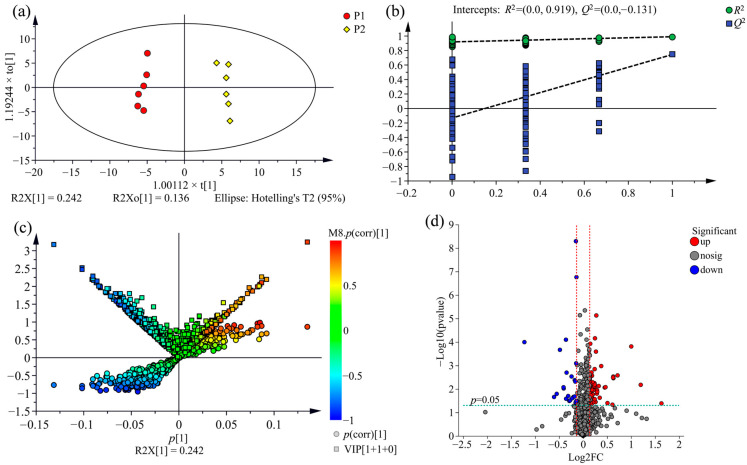
Differential metabolites analysis for the comparison group of P1 vs. P2 using OPLS-DA. (**a**) OPLS-DA score plot (R2X = 0.379, *R*^2^ = 0.990, *Q*^2^ = 0.745). (**b**) Permutation test plot with 200 iterations. (**c**) OPLS-DA (V + S)-plot, the potential differential metabolites situated on the lower left and upper right end of the “S”, the same are the upper of both side of the “V”, the deeper the colour of dots the more important metabolites. (**d**) Volcano plot. Each dot represents an identified metabolite; the blue dots on the left, the red dots on the right and the grey dots on the middle of diagram indicate down-regulated, up-regulated and insignificant differential metabolites, respectively.

To explore the variation of metabolic profile across the wood of three clones further, an OPLS-DA model was performed to determine differential metabolites between the comparison groups from three clones investigated, namely, P1 vs. P3, P2 vs. P3, P1 vs. P2. As shown in Figure 4a, Figure 5a and Figure 6a, the comparison group of samples that belong to the three clones were obviously divided into distinct chemotype-cluster according to their metabolite profiles, and the separation of the clone samples was based on the genetic background, which demonstrates that the variation of metabolic profiles across the wood of three clones is correlated for the discrimination among variety.

To evaluate the overfitting possibility of the OPLS-DA model, permutation testing with 200 iterations was performed. In theory, all the values of *R*^2^ and *Q*^2^ for the model fitting (on the left) are below the points of the original model (on the top-right corner), and the regression line of the *Q*^2^-point intersects the vertical axis on the left at zero or less, indicating that the original model is valid [36]. As shown in Figure 4b, Figure 5b and Figure 6b, the permutation plots of all comparison groups display that the models are satisfactory and could be applied for further analysis of the metabolite variation.

### 2.4. Comparison of Metabolic Variations between Three Clones

To screen statistically differential metabolites, a (V + S)-plot was constructed from the OPLS-DA model. As shown in Figure 4c, Figure 5c and Figure 6c, the variables with variable importance in the projection (VIP) ≥ 1 had higher *p* and *p* (corr) values, which are generally situated on the lower left and upper right end of the “S”, and were usually regarded as the potential differential metabolites associated with distinguishing the comparison groups. There are 205 potential differential metabolites between the comparison group of P1 and P3, 212 between P2 and P3, and 251 between P1 and P2 were screened out according to VIP ≥ 1 and *p* < 0.05.

Additionally, volcano plots were constructed to visualise the significantly differential metabolites in the comparison group. As shown in Figure 4d, Figure 5d and Figure 6d, the dots located farther to the left, right side and top of the volcano plot represent greatly differential metabolites. By adding the threshold of fold-change score of ≥1.1 (up-regulated) or ≤0.9 (down-regulated), the significantly differential metabolites were screened in the comparison groups.

**Figure 7:**
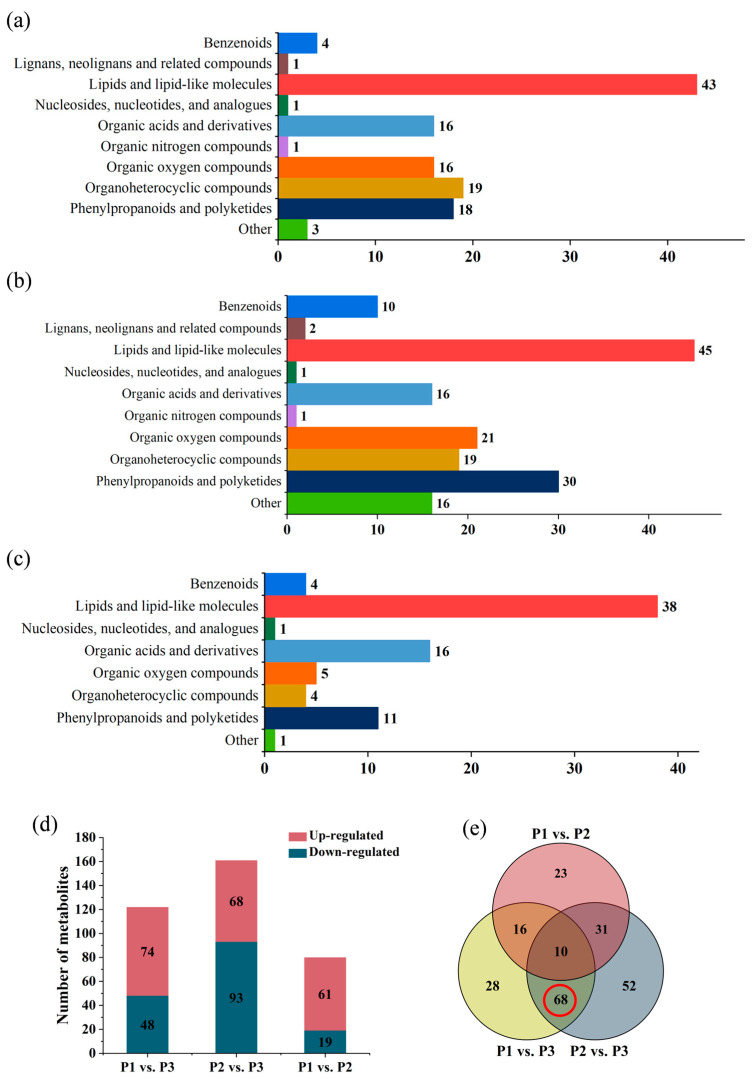
Analysis of differential metabolites data for the three comparison groups. Classification of the significant differential metabolites between the comparison groups of (**a**) P1 vs. P3; (**b**) P2 vs. P3; (**c**) P1 vs. P2. (**d**) The number of differential metabolites between P1 vs. P3, P2 vs. P3, P1 vs. P2, respectively. Up-regulated differential metabolites were represented red pillars; down-regulated differential metabolites were represented blue pillars. (**e**) The Venn diagram of differential metabolites of the comparison groups P1 vs. P3, P2 vs. P3, P1 vs. P2, respectively.

Based on the comparison between P1 and P3, 122 significantly differential metabolites were identified, including 43 lipids and lipid-like molecules, 19 organoheterocyclic compounds, 18 phenylpropanoids and polyketides, 16 organic acids and derivatives, 16 organic oxygen compounds, 4 benzenoids and 6 others (Figure 7a), among which 48 metabolites display down-regulated and 74 metabolites display up-regulated in P1. The comparison between P2 and P3 revealed that 161 metabolites were significantly different, and they contain 45 lipids and lipid-like molecules, 30 phenylpropanoids and polyketides, 16 organic acids and derivatives, 21 organic oxygen compounds, 19 organoheterocyclic compounds, 10 benzenoids and 20 others (Figure 7b). Among the 161 differential metabolites, there were 93 down-regulated metabolites, and 68 up-regulated metabolites in clone P2. Furthermore, based on the comparison between P1 and P2, 80 significantly differential metabolites (19 down-regulated and 61 up-regulated in clone P1) were found, covering 38 lipids and lipid-like molecules, 16 organic acids and derivatives, 11 phenylpropanoids and polyketides, 5 organic oxygen compounds, 4 benzenoids, 4 organoheterocyclic compounds and 2 others (Figure 7c). The differences of metabolites profiles among the three clones were shown in these results, which may be correlated to the genetic diversity between different clones.

As shown in Figure 7d, the number of differential metabolites were fewer in the *P. deltoides* comparison group (P1 vs. P2) than in the two other comparison groups (P1 vs. P3, P2 vs. P3), illustrating that the difference of metabolites profile was relatively smaller within the individual species considering their similar genetic backgrounds, while a more significant difference was observed between the two species. Therefore, analysis of metabolic variations might be useful for the distinctions of clone or species within genus *Populus*.

### 2.5. Screening of Potential Markers between P. nigra and P. deltoides

Venn diagram analysis was conducted to visualise the overlap and distinctions of differential metabolites among the three comparison groups of *Populus*, and 78 commonly differential metabolites were discovered between P1 versus P3 and P2 versus P3, including 68 metabolites with significant differences that may be related to the inter-specific difference within genus *Populus* (Figure 7e).

**Figure 8:**
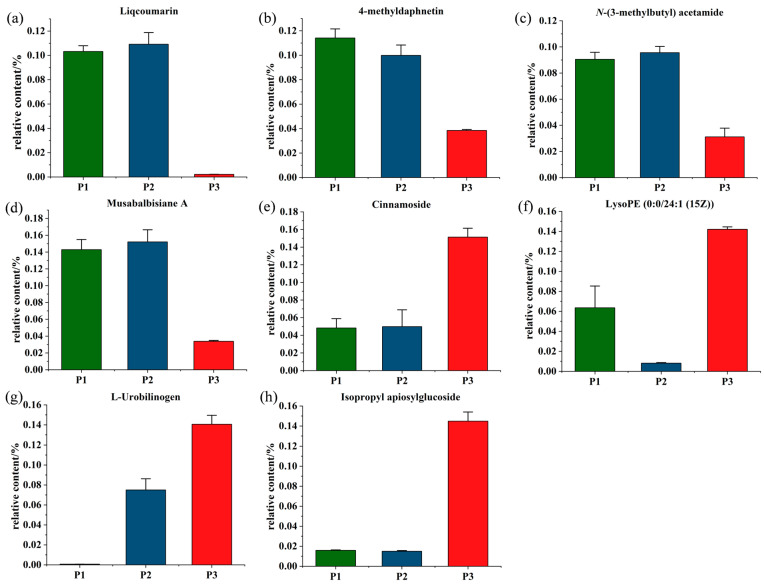
The relative abundance of the eight significantly differential metabolites between *P. nigra* and *P. deltoides*. (**a**) Liqcoumarin; (**b**) 4-methyldaphnetin; (**c**) *N*-(3-methylbutyl) acetamide; (**d**) Musabalbisiane A; (**e**) Cinnamoside; (**f**) LysoPE (0:0/24:1 (15Z)); (**g**) L-urobilinogen; (**h**) Isopropyl apiosylglucoside.

Based on the analysis of significant differential metabolites among species screened in the comparison groups, the screened metabolites were primarily involved in three superclasses, including lipids and lipid-like molecules, organoheterocyclic and organic oxygen compounds, and phenylpropanoids and polyketides. Altogether, compared with *P. nigra*, eight significantly changed metabolites were screened out in *P. deltoides*. As shown in Figure 8, four significantly differential metabolites were significantly increased, including liqcoumarin, 4-methyldaphnetin, *N*-(3-methylbutyl) acetamide and musabalbisiane A; while the other four were significantly reduced, including cinnamoside, lysoPE (0:0/24:1 (15Z)), L-urobilinogen and isopropyl apiosylglucoside. Those significant metabolites were chosen as the metabolic difference markers to separate *P. nigra* and *P. deltoides*.

### 2.6. Screening for the Differential Metabolites in the Three Clones of Poplar

To estimate the contribution of metabolites to distinction between the three investigated clones, one-way analysis of variance (ANOVA) was performed, where a *p*-value of <0.05 was considered significant, and 352 metabolites with significant correlation were obtained among P1, P2 and P3. Lipids, amino acids and carbohydrates are three categories of important metabolites that dominate the classification of the wood samples from different poplar clones, making up approximately 50% of 352 metabolites.

**Figure 9:**
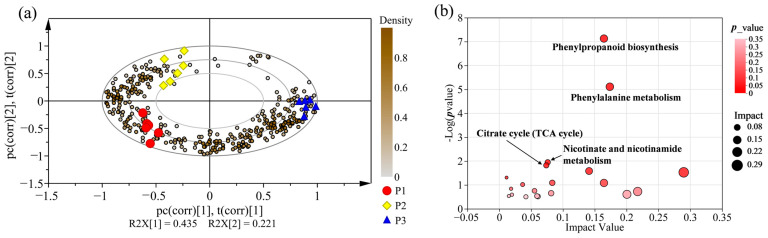
Preliminary analysis of discriminant metabolites data of three clones. (**a**) PCA /biplot of all samples constructed using 352 discriminant metabolites of three clones. The colour depth of dot represents the relative content in bases of the discriminant metabolites, and when the different metabolites situated near the respective clone cluster in the score plot, they were considered to be highly discriminant. (**b**) The metabolic pathways assignment of differential metabolites from three clone woods. Y-axis represents the logarithmic transformation of *p* values estimated by enrichment analysis. X-axis show the pathway impact value was calculated by topology analysis. Different sizes of bubbles represent impact value.

To further screen the metabolites that contributed the greatest to the distinction between clones, a biplot was constructed based on the dataset that includes 18 samples and 352 different metabolites, which were significantly correlated with variation between the three investigated clones (Figure 9a). The biplot could simultaneously display and illuminate the relationship among PCA scores and loadings, indicating which variable mainly determined the position of the samples in the PCA. Figure 9a shows the composition and contents of different metabolites among three clones in a biplot, which were clearly clustered into three distinct groups, thus improving the explained variances (43.5% for PC-1 and 22.1% for PC-2). Subsequently, the discriminant capacity of these metabolites was ranked according to the contribution to distinction between clones. The top 30 metabolites and their correct classification are shown in Table 1. These metabolites were highly discriminant metabolites between the poplar clones, containing ten lipids, five organic oxygen compounds, five organic acids and derivatives, four phenylpropanoid and polyketides, four benzenoids, one organoheterocyclic compound, and one nucleosides, nucleotides, and analogues.

### 2.7. Metabolic Pathway Enrichment Analysis for Differential Metabolites

To characterize the metabolic pathway of the differential metabolites that can discriminate different poplar clones, the Kyoto Encyclopaedia of Genes and Genomes (KEGG) pathway enrichment analysis was performed. A total of 352 metabolites were mainly enriched in four metabolic processes related to the biosynthesis of phenylpropanoid, phenylalanine metabolism, citrate cycle (TCA cycle) and nicotinate and nicotinamide metabolism (Figure 9b). The biosynthesis of phenylpropanoid displayed the highest score in the four metabolic pathways and contained some differential metabolites including caffeic acid, trans-cinnamic acid, sinapyl alcohol, l-phenylalanine, ferulic acid, cinnamaldehyde, *cis*-β-d-glucosyl-2-hydroxycinnamate and 4-hydroxycinnamyl aldehyde.

## 3. Discussion

Woody tissue, a unique component to ligneous plants, consists primarily of cellulose, hemicellulose, lignin, and extractives, and contributes significantly to a wide range of important physiological functions in tree, such as structural support, water and nutrient transport, reserve substances storage, and defence to pathogen infections and herbivore attacks [37]. The metabolites, as end-products of cellular regulatory processes and the key to the growth and development of trees, have levels influenced by environmental conditions and genetic variance [3,5,6,7,21,38]. These factors may change the biosynthetic pathways of metabolites, which regulates the relative abundance and composition of metabolites [39].

Understanding of the influence of genetic background on metabolites of wood is of important significance in tree improvement and exploring the variability trends of wood characteristic in species with long harvest periods. At present, the information in regard to the relevance of genetic background on metabolic variation among different cultivars is scarce. In the few studies of this kind, the most common platform used for analysis was GC-MS [21,35,40].

In this work, the components and variation of metabolites in the wood of one *P. nigra* (P3) and two *P. deltoides* clones (P1 and P2) in poplar were investigated using UPLC-Triple-TOF-MS with non-targeted metabolic analysis, in order to detect significant genetic variation for metabolites and understand if the different poplar genotypes can be distinguished according to their metabolites, and if so, which metabolites are the marker of the distinction.

P1, P2 and P3 are all important cultivars of forest poplar in China, and vary in growth and wood properties [38,41,42]. The variety, P2, is an intraspecific hybrid that was bred by artificial pollination with P1 as the female parent and *P. deltoides* CL. ‘2KEN8’ as the male parent [43]. Compared to its parents, P2 is superior to other poplar species in terms of growth and wood traits, including faster growth rate, straighter trunk, and stronger resistance against pests [44]. P1 had better physical and mechanical properties than P3, as a previous study has shown [45]. In this research, no difference in age and growing conditions existed among the three clones as they came from the same forest farm and were simultaneously grown and cut. The effect of the genotype itself on the metabolomics profile of three poplar clones wood is revealed. In that sense, the unique metabolic properties of different clones that were obtained in this study could be applied to define the difference of genetic backgrounds among them.

In three clones, a total of 699 metabolites were obtained. A large amount of information about metabolites of poplar wood provides the foundation for the isolation and further development and utilization of valuable components.

PCA and HCA made from the variation of the metabolite abundance in the different poplar clones allowed the initial distinction of the metabolite profile in three clusters, consistent with the initial classification of the samples conducted on their intra-specific diversity that might be caused different clones or varieties in a same species [38,41,42]. Moreover, the farther distinction between P1, P2 and P3 samples is also consistent with the most frequently used classification of the various clones of genus poplar into different species [46], which places P3 under *P. nigra* and P1 and P2 under *P. deltoides*. Therefore, the PCA and HCA reveals the composition and relative abundance of the wood metabolites of three poplar clones that can be used for chemotaxonomic purposes.

Herein, two phenylpropanoids and polyketides, two lipids and lipid-like molecules, one organic acid and derivative, one diterpenoid, one organoheterocyclic compound, and one organic oxygen compound, all of which display significant variation in *P. nigra* and *P. deltoides* comparisons, were screened out. These significantly changed metabolites included liqcoumarin, 4-methyldaphnetin, *N*-(3-methylbutyl) acetamide, musabalbisiane A, cinnamoside, lysoPE (0:0/24:1 (15Z)), L-urobilinogen and isopropyl apiosylglucoside. Of these changed metabolites the first four metabolites were remarkably increased and the other four metabolites were significantly reduced between *P. deltoides* and *P. nigra*. The composition and abundance of these different metabolites between two species of poplar indicated their species-specific significance and may be considered as chemotaxonomic markers for the discrimination of the poplar species.

All of the metabolites were chosen for ANOVA to further interrogate the chemotype distinction of genotypically differentiated clones of the hybrid poplar. A total of 352 metabolites with ANOVA *p*-value below 0.05 facilitated the separation of the three clones, and were dominated by a disproportionate number of lipids, carbohydrates and amino acids. Guerra et al. [21] used GS-TOF-MS to analyse the genetic variation in the wood metabolomic composition of different *Populus trichocarpa* clones, finding 11 statistically significant (*p* < 0.01) different metabolites. A total of 197 metabolites with significant differences were identified between the two multi-gene transgenic and one nontransgenic lines of hybrid poplar, in which the three primary metabolites, amino acids, lipids and carbohydrates, constituted up to 70% of all identified metabolites [22].

Among these samples, 352 metabolites that were significantly correlated with variation between the three investigated clones were mainly assigned to four metabolic pathways associated with the biosynthesis of phenylpropanoid, phenylalanine metabolism, TCA cycle and nicotinate and nicotinamide metabolism. Previous research has found that pathways phenylpropanoid biosynthesis, the citrate cycle and phenylalanine metabolism were associated with plant defence [5,9,47]. The biosynthesis of phenylpropanoid, which enriched some differential metabolites containing caffeic acid, *trans*-cinnamic acid, sinapyl alcohol, l-phenylalanine, ferulic acid, cinnamaldehyde, *cis*-β-d-glucosyl-2-hydroxycinnamate and 4-hydroxycinnamyl aldehyde, was the most significant enriched pathways (*p* < 0.05) in four metabolic pathways.

The biosynthesis of phenylpropanoid is the main synthetic pathway of secondary metabolite in plants, plays an important role in resistance to biotic stress [48]. For instance, phenylalanine, as the entry point to the pathway of phenylpropanoid biosynthesis, was transformed into trans-cinnamic acid through the catalysis of phenylalanine ammonia-lyase. Then, according to differences of the plant species, benzoic acid 2-hydroxylase converts *trans*-cinnamic acid to salicylic acid via the intermediates ortho-coumaric acid or benzoic acid [49,50]. Salicylic acid, a critical plant hormone, can be direct or indirect to regulate many aspects of plant growth and development, and influence resistances to (a)biotic stresses in plants [51]. Sinapyl alcohol, which was involved in the biosynthesis of phenylpropanoid, was the precursor of lignin biosynthesis. The deposition of lignin generates a highly thickened secondary cell wall of xylem vessels and fibres to result in the formation of wood [52]. The thickened secondary cell wall of xylem is difficult to feed and digest for insects. In addition, four differential metabolites were substantially enriched in the biosynthesis of phenylpropanoid (caffeic acid, *trans*-cinnamic acid, sinapyl alcohol, and ferulic acid) were associated with chemical defence in poplar. The accumulation of ferulic acid was induced in xylem of poplar clone P2 by *Apriona germari* (Hope) indicated ferulic acid might be involved in plant defence against stem-boring insects [9]. It was found that four hydroxycinnamates, benzenoids and their derivatives (caffeic acid, trans-cinnamic acid, sinapyl alcohol and coumarin) contribute constitutive resistance to fall webworm (*Hyphantria cunea*) in *P. deltoides* [35]. It was indicated that the pathway to phenylpropanoid in poplar play an important role in the responses to biotic stresses in *Populus*.

## 4. Materials and Methods

### 4.1. Wood Material Collection

The wood samples from eight-year-old poplar clones (6.5–8.0 m above the ground; i.d., approximately 20 cm) of *P. deltoides* CL. ‘55/65’ (P1), *P. deltoides* CL. ‘Danhong’ (P2) and *P. nigra* CL. ‘N179’ (P3) were collected in December 2017 from Kaihua County in Zhejiang province of China. The three clones belong to different species of Sect. *Aigeiros* Duby, where P1 and P2 both are *P. deltoides* W. Bartram ex Marshall and P3 is *Populus nigria* L. Six biological replicates were performed for each clone. The naturally dried samples were ground into powder and placed aside.

### 4.2. Metabolite Extraction

Approximately 800 µL of methanol/water (4:1, *v*/*v*) extractant and 20 µL of internal standards (0.3 mg/mL 2-chloro-L-phenylalanine in acetonitrile) was added into 50 mg powder of each sample. The sample tissue was broken using high-throughput tissue crusher (Wonbio-96c, Shanghai Wanbo Biotechnology Co., Ltd., Shanghai, China) at −20 °C and 50 Hz for 6 min, and then treated for 30 min by ultrasound (SBL-10DT, Ningbo Xinzhi Biotechnology Co., Ltd., Ningbo, China) at 5 °C at 40 kHz to extract metabolites. After precipitation at −20 °C for 30 min, centrifugation (Centrifuge 5430R, Eppendorf, Hamburg, Germany) was performed on the samples at 13,000× *g* for 15 min at 4 °C. The obtained supernatant was absorbed, dried, and dissolved in 150 µL of acetonitrile: water (4:1, *v*/*v*) solution for further analysis.

### 4.3. Quality Control Sample

To assess the stability of the analysis, the quality control sample (QC) was prepared by mixing equal volumes of all sample extracts. During the analytic sequence, the QC samples were measured at regular intervals in the same manner as the wood extracts.

### 4.4. Profiling of Metabolites by UPLC-Triple-TOF-MS

Metabolite profiling of poplar wood was conducted by using a UPLC-Triple-TOF-MS analytical platform based on UPLC with an ExionLC^TM^AD system (Applied Biosystems, Waltham, MA, USA) equipped with an ACQUITY UPLC BEH C18 column (100 mm × 2.1 mm × 1.7 µm, Waters, Milford, MA, USA), and a quadrupole-time-of-flight mass spectrometer (Triple TOF^TM^ 5600+, Applied Biosystems) fitted with an electrospray ionization (ESI). Water with 0.1% formic acid was used as mobile phase A, while the mobile phase B comprised acetonitrile: isopropanol (1:1, *v*/*v*) solution with 0.1% formic acid. The following was gradient elution program: 95% (A):5% (B) at 0 min, 80% (A):20% (B) at 3 min, 5% (A):95% (B) at 9 min, 5% (A):95% (B) at 13 min, 5% (A):95% (B) at 13.1 min, and 95% (A):5% (B) at 16 min, 95% (A):5% (B) for equilibrating the systems. The temperature of chromatographic column, flow rate and injection volume of each sample extract were set at 40 °C, 0.4 mL/min and 20 μL, respectively. During the period of analysis, all these samples were stored at 4 °C.

The extract sample of poplar wood was analysed in positive mode (POS) and negative mode (NEG) over a mass range of 50–1000 *m*/*z*. The MS conditions were set as follows: temperature of ESI source, 500 °C; ion-spray voltage floating in positive mode and negative mode was 5.00 and −4.00 kV, respectively; both ion source gas 1 and gas 2 was 50 psi; curtain gas at 30 psi; declustering potential at 80 V; and collision energy was 20–60 V rolling for MS/MS.

### 4.5. Metabolite Identification

The obtained raw data were pre-processed in Progenesis QI 2.3 (Waters), and the procedure included baseline correction, peak detection and retention time alignment. The processed data matrix that was composed of peak intensity, retention time, characteristic fragment and mass-to-charge ratio (*m*/*z*) values was generated.

Structural annotation of metabolites was performed through the accurate *m*/*z* values, associated adducts and MS/MS characteristic fragmentation data with automatically matching in credible biochemical databases, including HMDB and Metlin database.

### 4.6. Statistical Analysis

After the raw metabolic data were normalized and imputed as previously described in Yang et al. [53], multivariate statistical analysis was carried out on log-transformed data by using SIMCA-P 14.1 software package (Umetrics, Umeå, Sweden). Two unsupervised pattern recognition methods, PCA and HCA, and OPLS-DA were performed. Consequently, statistically significant metabolites that helped in distinguishing the clones of poplar were screened according to a fold-change score of ≥1.1 or ≤0.9, *p*-value < 0.05, and VIP value of ≥1 in the OPLS-DA model. ANOVA was conducted to select the highly discriminant metabolites among three investigated clones. Metabolic pathway annotation analysis was performed to obtain the significant pathways of the differential metabolites base on the KEGG database [54].

## 5. Conclusions

In summary, the metabolites in the wood of one *P. nigra* and two *P. deltoides* clones in poplar were investigated using UPLC-Triple-TOF-MS. HCA and PCA suggested that the metabolic differences that were obtained in the wood composition have allowed distinguishing poplar species, which is consistent with the initial classification of the specimens conducted from the morphological features and crossability encounters.

Through conducting OPLS-DA and Venn analysis, the compounds including liqcoumarin, 4-methyldaphnetin, *N*-(3-methylbutyl) acetamide, musabalbisiane A, cinnamoside, lysoPE (0:0/24:1 (15Z)), L-urobilinogen and isopropyl apiosylglucoside were identified as signature differential metabolites of *P. nigra* and *P. deltoids*, which may be considered as valuable markers for chemotaxonomy.

In addition, remarkable difference in the expression level of 352 metabolites was observed between the three clones, and those may be closely related to the distinction in unique properties (e.g., growth, rigidity and tolerance) of the poplar wood cultivars. At the same time, the use of composition and abundance of the metabolites of these three poplar clones can achieve chemotaxonomic purposes. The findings of this study may provide the foundation for further research on wood metabolism in poplar, meanwhile the chemotaxonomic markers were obtained in this study will stimulate the early screening of potentially superior tree in molecular breeding of poplar clones based on inhere wood quality traits.

## Figures and Tables

**Table 1:** The top 30 metabolites that contributed significantly to the correct classification of P1, P2 and P3.

Metabolite	*p*_Value	FDR	A	B	C	Superclass
*cis*-3-Hexenyl hexanoate	6.85 × 10^−5^	1.58 × 10^−3^	1.888	1.841	2.277	a
3-hydroxy-3-methyl-Glutaric acid	1.00 × 10^−2^	3.46 × 10^−2^	3.059	3.030	3.331	a
(−)-11-Hydroxy-9,15,16-trioxooctadec-anoic acid	4.03 × 10^−3^	1.93 × 10^−2^	3.848	3.698	3.723	a
Myristoleic acid	1.28 × 10^−4^	2.24 × 10^−3^	3.026	3.079	3.265	a
Alpha-Linolenic acid	3.61 × 10^−2^	6.68 × 10^−2^	3.574	3.273	3.243	a
1,11-Undecanedicarboxylic acid	4.20 × 10^−4^	4.64 × 10^−3^	2.931	2.976	3.182	a
Capsidiol	7.49 × 10^−4^	6.61 × 10^−3^	2.032	2.007	2.231	a
Furanofukinin	1.05 × 10^−2^	3.56 × 10^−2^	2.580	2.800	2.515	a
Stachyoside A	3.18 × 10^−3^	1.67 × 10^−2^	3.474	3.434	3.636	a
Sorbitan laurate	4.19 × 10^−2^	9.08 × 10^−2^	2.687	2.604	2.562	a
L-Phenylalanine	3.99 × 10^−4^	3.44 × 10^−3^	0.059	0.056	0.108	b
Neuromedin B (1-3)	5.32 × 10^−3^	2.33 × 10^−2^	3.406	3.579	3.343	b
6-Oxopiperidine-2-carboxylic acid	3.36 × 10^−2^	7.80 × 10^−2^	1.957	2.159	1.976	b
Glutamylglycine	2.32 × 10^−2^	4.82 × 10^−2^	2.284	1.652	1.584	b
3-Hydroxy-L-proline	7.23 × 10^−3^	2.15 × 10^−2^	2.540	2.296	2.270	b
Natamycin	5.18 × 10^−4^	3.91 × 10^−3^	2.330	2.576	2.342	c
Glucosylisomaltol	1.24 × 10^−3^	6.98 × 10^−3^	2.177	2.106	2.513	c
6-(4-ethyl-3-hydroxyphenoxy)-3,4,5-trihydroxyoxane-2-carboxylic acid	1.31 × 10^−2^	4.12 × 10^−2^	2.592	2.725	2.542	c
Kinetin-7-*N*-glucoside	2.95 × 10^−3^	1.19 × 10^−2^	3.433	3.357	3.341	c
3-Hydroxy-1-(4-hydroxyphenyl)-1-propanone	9.00 × 10^−3^	3.25 × 10^−2^	2.510	2.653	2.496	c
Tipranavir	1.18 × 10^−4^	2.15 × 10^−3^	2.677	2.583	3.065	d
Oxacyclotetradecan-2-one	5.95 × 10^−3^	2.50 × 10^−2^	1.759	1.841	2.226	d
6″-O-Acetylglycitin	4.92 × 10^−2^	8.43 × 10^−2^	2.401	2.231	2.221	d
Caffeic Acid	4.64 × 10^−2^	8.10 × 10^−2^	2.856	2.641	2.662	d
Hovenolactone	1.98 × 10^−3^	1.24 × 10^−2^	2.909	3.002	3.406	e
Benzoic acid	3.36 × 10^−2^	6.32 × 10^−2^	1.664	1.463	1.488	e
4-ethenyl-6-methoxybenzene-1,3-diol	3.24 × 10^−2^	7.58 × 10^−2^	3.365	3.451	3.337	e
2′-Hydroxyacetophenone	9.17 × 10^−3^	3.29 × 10^−2^	1.854	2.075	1.805	e
1-(4-methoxy-1-benzofuran-5-yl) ethan-1-ol	3.38 × 10^−2^	6.34 × 10^−2^	2.040	1.856	1.778	f
5-Methyldeoxycytidine	5.76 × 10^−5^	9.70 × 10^−4^	3.910	3.627	4.415	g

The *p*-value and FDR indicate the significance and false discovery rate of metabolites difference among three clones, respectively. A, P1_mean; B, P2_mean; C, P3_mean. a, lipids and lipid-like molecules; b, organic acids and derivatives; c, organic oxygen compounds; d, phenylpropanoids and polyketides; e, benzenoids; f, organoheterocyclic compounds; g, nucleosides, nucleotides, and analogues.

## Data Availability

The data presented in this study are available on request from the corresponding author.

## Supplementary Materials

The following supporting information can be downloaded at: https://www.mdpi.com/article/10.3390/molecules28207024/s1, Figure S1: The stacking plot of total ions current maps, Table S1: Metabolites of poplar wood from three clones.

## Sample Availability

Samples of the compounds are not available from the authors.
