# Supplementary figures and images for: Comparative Analysis of the Wood Metabolites of Three Poplar Clones Using UPLC-Triple-TOF-MS
Source: Molecules. 2023 Oct 11;28(20):7024. doi: 10.3390/molecules28207024 (PMC10609545; doi:10.3390/molecules28207024)

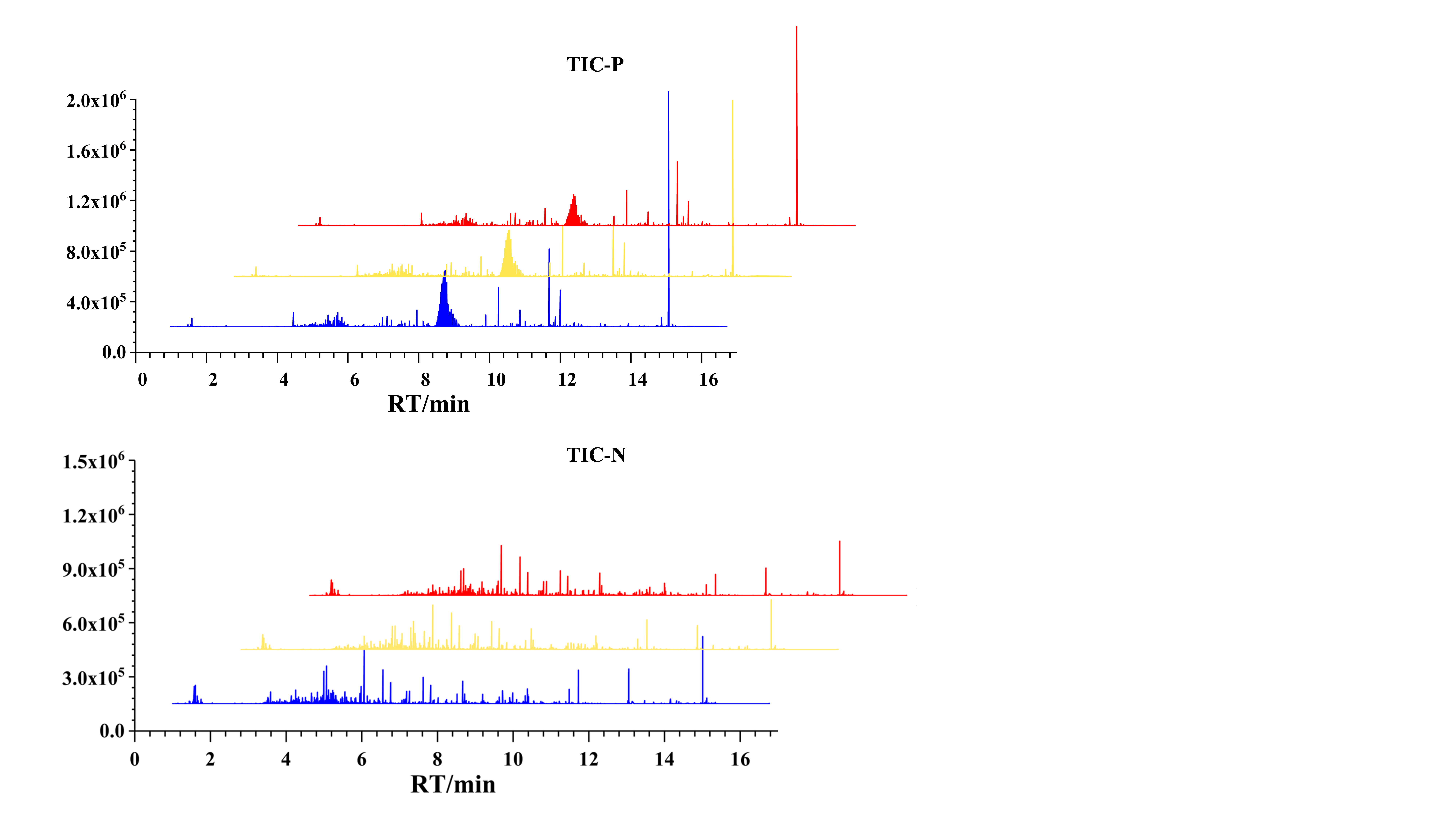

Supplement: Supplementary file 1 [file molecules-28-07024-s001.zip › supplementary/Supplementary Materials Figure S1 (The stacking plot of total ions current maps).tif]
